# Insulin resistance predicts progression of de novo atherosclerotic plaques in patients with coronary heart disease: a one-year follow-up study

**DOI:** 10.1186/1475-2840-11-71

**Published:** 2012-06-18

**Authors:** Xuanqi An, Dong Yu, Ruiyan Zhang, Jinzhou Zhu, Run Du, Yuhang Shi, Xiaowei Xiong

**Affiliations:** 1 Department of Cardiology, Ruijin Hospital, Jiaotong University School of Medicine, Shanghai, 200025, People's Republic of China

**Keywords:** Coronary heart disease, Insulin resistance, Atherosclerosis plaque progression, In-stent restenosis, one-year follow-up, HOMA-IR

## Abstract

**Background:**

The aim of our study was to explore and evaluate the relationship between insulin resistance and progression of coronary atherosclerotic plaques. With the great burden coronary heart disease is imposing on individuals, healthcare professionals have already embarked on determining its potential modifiable risk factors in the light of preventive medicine. Insulin resistance has been generally recognized as a novel risk factor based on epidemiological studies; however, few researches have focused on its effect on coronary atherosclerotic plaque progression.

**Methods:**

From June 7, 2007 to December 30, 2011, 366 patients received their index coronary angiogram and were subsequently found to have coronary atherosclerotic plaques or normal angiograms were consecutively enrolled in the study by the department of cardiology at the Ruijin Hospital, which is affiliated to the Shanghai Jiaotong University School of Medicine. All patients had follow-up angiograms after the 1-year period for evaluating the progression of the coronary lesions. The modified Gensini score was adopted for assessing coronary lesions while the HOMA-IR method was utilized for determining the state of their insulin resistance. Baseline characteristics and laboratory test results were described and the binomial regression analysis was conducted to investigate the relationship between insulin resistance and coronary atherosclerotic plaque progression.

**Results:**

Index and follow-up Gensini scores were similar between the higher insulin lower insulin resistant groups (9.09 ± 14.33 vs 9.44 ± 12.88, *p* = 0.813 and 17.21 ± 18.46 vs 14.09 ± 14.18, *p* =0.358). However the Gensini score assessing coronary lesion progression between both visits was significantly elevated in the higher insulin resistant group (8.13 ± 11.83 versus 4.65 ± 7.58, *p* = 0.019). Multivariate logistic binomial regression analysis revealed that insulin resistance (HOMA-IR > 3.4583) was an independent predictor for coronary arterial plaque progression (OR = 4.969, *p* = 0.011). We also divided all the participants into a diabetic (n = 136) and a non-diabetic group (n = 230), and HOMA-IR remained an independent predictor for atherosclerosis plaque progression.

**Conclusions:**

Insulin resistance is an independent predictor of atherosclerosis plaque progression in patients with coronary heart disease in both the diabetic and non-diabetic population.

## Background

Although drug-eluting stents prevail in treating coronary heart disease (CHD), several concerns have already begun to rise publicly, including those regarding medical complications social burdens [1]. More cost-effective preventive treatments are called for; therefore, various studies have been conducted to identify the risk factors for CHD, which are classified into classical versus novel categories [2]. The former consists of aging, female sex, genetic factors, obesity, smoking, dyslipidemia, hypertension and diabetes mellitus while the latter includes several inflammatory markers such as hsCRP, IL-6, TNF-α, PAI-1, MCP-1 and adhesion factors such as ICAM-1 and VCAM-1 [2]. Some studies also divert their targets on the specific group of patients such as patients with hypertension, diabetes, metabolic syndrome or even SLE to personalize the existing grading systems of risk factors including the classic Framingham, Reynolds, SCORE and ASSIGN [2]. Insulin resistance, frequently appears in various clinical settings such as hypertension, diabetes and metabolic syndrome and is believed to be responsible for connecting endocrinological disorders with their potential adverse cardiovascular complications based on its reciprocal relationship with endothelial dysfunction indicated by cellular, physiological, clinical, and epidemiological studies [3-5]. However, few studies have actually examined its relationship with the progression of coronary atherosclerotic plaques. The aim of our study was to explore the effect of insulin resistance on the progression atherosclerotic plaques in patients with CHD.

## Methods

Since June 7^th^, 2007 to Dec 30^th^, 2011, subjects were identified and screened from patients seeking a planned percutaneous intervention for suspected new onset of CHD based on the clinical presentation, which was categorized as ECG abnormality (only positive stress ECG), stable angina (stable exertional symptoms only), unstable angina (progressive symptoms or symptoms at rest), or MI (creatinine kinase [CK] level >636 IU/L and creatinine kinase-myocardial band isoenzyme [CK-MB] index >6%) in the catheter Lab at the Shanghai Ruijin Hospital which is affiliated with the Shanghai Jiaotong University School of Medicine . Written consent was obtained from all individuals before they were enrolled in the study. Their medical histories were obtained and recorded, and pertinent baseline laboratory studies were drawn before cardiac catheterization while BMI and GFR were calculated. All patients were treated with IV heparin and combined antiplatelet therapy, while the use of IIb/IIIa inhibitors was at the operator’s discretion. Telephone interviews were performed at 6 months and participants were readmitted for routine angiographic follow-up 1 year after the procedure, including those with normal angiography at their index visits. Necessary cardiac catheterization was performed for recurrent symptoms or objective evidence of ischemia. Individuals were excluded from the study if they fell into one of the following criteria: Patients with histories of CHD or prior coronary revascularization; patients undergoing CABG instead of stenting; patients with heart failure or any kind of cardiomyopathy; patients with familial hypercholesterolemia; patients with severely impaired liver or renal function; patients with a terminal illness with a life expectancy of less than 1 year.

### Laboratory studies and the assessment of insulin resistance

After fasting for 12 hours, blood samples were drawn from the patients at 8 AM, including both fasting glucose and insulin concentration, HbA1c, hsCRP, liver function tests, lipid panel, BUN, creatinine and urine acid. In addition, OGTT and insulin concentrations were also obtained 2 hours after oral ingestion of 75 mg glucose by the central lab in the hospital without the knowledge of the study. Body mass index (BMI) was calculated as weight divided by height squared (kg/m^2^). We estimated insulin resistance by using the homeostasis model assessment index of IR (HOMA-IR) developed by Mathew [6], which was believed to have a close correlation with euglycemic clamp for use in cross-sectional studies [7]. We used the following formula HOMA-IR = baseline insulin concentration (U/mL) × baseline glucose concentration (mmol/L)/ 22.5 [6]. We obtained HOMA-IR values from 284 individuals and subsequently defined the cutoff value for insulin resistance as the upper quartile of HOMA-IR obtained from all the subjects involved in the study [8], which equals 3.458 in our study. Also glomerular filtration rate (GFR) was calculated by adopting the Cockcroft-Gault equation (for men: (140 − age × weight)/(72 × serum creatinine); for women: (140 − age × weight) / (72 × serum creatinine × 0.85)) [9].

### Coronary angiography and Gensini score

**Figure 1:**
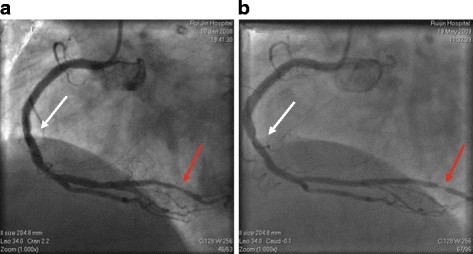
**Illustration of initial and follow-up visit angiograms obtained from one single participant.** Figure 1a depicts the index angiogram while Figure 1b is the follow-up angiogram from the same angle. To visualize the coronary lesions progression, two sets of colored arrows were presented to manifest different lesions. In addition, both lesions progressed during the 1 year follow-up. Figures were obtained with permission from the catheter lab at the Shanghai Ruijin Hospital, affiliated to Shanghai Jiaotong University School of Medicine.

Standard method was adopted and Judkins method was deployed. All patients were admitted to the hospital the day before CAG. The purpose of CAG was to diagnose any ischemic conditions such as angina. After administration of isosorbide dinitrate (2.5–5 mg bolus dose), the coronary arteries were evaluated by 2 experienced cardiologists. Narrowing of ≥50% in one of the three major coronary arteries or their major branches was judged significant while narrowing of <5% was considered insignificant. We defined both residual narrowing of less than 20% and third class TIMI blood flow as successful outcomes of the procedure. The decision to deploy a balloon or use a specific drug-eluting stent was made by the cardiologists themselves. All angiograms were evaluated in similar angiographic angles, and the percentage of stenosis was determined with the use of calipers comparing the stenotic segment with the proximal, angiographically normal segment. Clodigrel, 75 mg per day, or ticlopidine, 250 mg twice per day, was administered for at least 12 months after the procedure and all patients received 100 mg aspirin per day. The same guidelines were implemented at 1-year follow-up for each subject. We also adopted the modified Gensini scoring system to evaluate both the baseline and the follow-up angiograms [10]. To summarize this scoring system, five points were given for left main lesion; 2.5 points for the proximal left anterior descending artery (LAD) or left circumflex (LCX); 1.5 points for mid-segment LAD; one point for the distal segment of LAD, first diagonal branch, LCX obtuse marginal branch or right coronary artery, and 0.5 points for the second diagonal branch or LCX posterolateral branch (Figure 1). To define the atherosclerotic plaque progression, we divided the following patients into the progression group: (1) patients who progressed from normal or insignificant angiogram to CAD; (2) Patients whose documented baseline lesions exacerbated in the same vessel; (3) Patients who progressed from their baseline CAD to new lesion(s) in a different vessel in one-year period. In addition, we classified those who had normal angiograms or similar CAD lesions at the first and following visits into the non-progression group [11].

### Definitions concerning conventional risk factors of CHD

Patients who had smoked in the past year were considered current smokers. The definition of hypertension was a blood pressure that was equal to or higher than 140/90 mmHg after three measurements during the first visit or those with previously established diagnosis of hypertension or who were receiving antihypertensive drugs. Individuals with at least two fasting plasma glucose levels higher than 125 mg/dL or those being treated for diabetes with oral hypoglycemic agents and/or insulin were considered diabetic. Patients were considered dyslipidemic if the individual had cholesterol levels higher than 200 mg/dL or triglyceride levels higher than 150 mg/dL or if they were receiving lipid-lowering drugs.

### Statistical analysis

We adopted SPSS11.0 for all the statistical analyses related to the study. Measurement data were expressed as mean and SD while count data were presented as percentage (%). We used the paired t-tests for continuous variables and the χ^2^ test or the Fisher exact for categorical variables. Nonparametric methods were preferred to handle non-normally distributed data. Several conventional and novel risk factors such as age, body mass index (BMI), hypertension, DM, LDL-C, HbA1c, hs-CRP, urine MA/Cr and HOMA-IR were included in the multivariate logistic regression model to determine their independent effects on the progression of atherosclerotic plaques. Differences were considered statistically significant when the *p* value was less than 0.05.

## Results

### Baseline demographics and lab results in the progression group versus non-progression group

A total of 377 patients were consecutively included during the 4-year period and 366 participants received their follow-up angiography, with 198 individuals included in the progression group (including119 patients with new lesions in different vessels and 134 patients with progression in the same vessel) and 168 in the non-progression group. Table 1 lists baseline demographic data for both groups. No significant difference can be seen between the two groups except for DM prevalence (42.9% versus 30.4%, *p* = 0.013), NGR prevalence (33.8% versus 51.2%, *p* = 0.001) and oral glucose lowering agent (35.9% versus 21.6%, *p* = 0.002). In addition, both initial clinical presentations and medications patients received at discharge were similar in the two groups.

**Table 1:** Baseline demographic data of the progression and the non-progression groups

**Variables**	**Progression Group** **(n = 198)**	**Non-progression Group (n = 168)**	*** p *value**
Age	65.83 ± 11.46	64.74 ± 10.19	0.375
Sex(Man/Women)	158/40	139/29	0.474
BMI (kg/m^2^)	25.47 ± 3.18	24.80 ± 3.63	0.421
Hypertension (%)	153 (77.3)	121 (72.5)	0.289
Diabetes (%)	85 (42.9)	51 (30.4)	0.013
NGR (%)	67 (33.8)	86 (51.2)	0.001
IFG (%)	13 (6.6)	7 (4.2)	0.362
IGT (%)	20 (10.1)	18 (10.8)	0.865
IFG + IGT (%)	13 (6.6)	6 (3.6)	0.241
Initial admission n (%)			
ECG abnormalities	24 (12.1)	12 (7.1)	0.111
Stable Angina	29 (14.6)	33 (19.6)	0.204
Unstable Angina	86 (43.4)	70 (41.7)	0.733
Non-ST elevated AMI	10(5.1)	9(5.4)	0.895
ST elevated AMI	49 (24.7)	44 (26.2)	0.752
Medication (%)			
Statin	196 (99.5)	165 (98.8)	0.468
CCB	67(29.6)	55(32.9)	0.806
ACEI/ARB	104 (53.6)	76 (46.1)	0.154
β-blocker	164 (82.8)	128(76.6)	0.141
Oral glucose lowering dug	70(35.9)	35(21.2)	0.002
Application of insulin	9(4.5)	11(6.5)	0.491

Table 2 compares the lab results between the two groups, revealing that the progression group had a worse performance than the non-progression group in the glucose panel including fasting glucose (6.15 ± 1.81 versus 5.49 ± 1.25, *p* <0.001 and 7.84 ± 1.80 versus 5.30 ± 1.22, *p* < 0.005), OGTT (10.09 ± 4.17 versus 8.65 ± 3.41, *p* < 0.001 and 9.68 ± 3.79 versus 8.47 ± 3.38, *p* = 0.002) and HbA1c (6.73 ± 1.40 versus 6.24 ± 0.98, *p* = 0.001 and 6.79 ± 1.53 versus 6.07 ± 0.78, *p* < 0.001). Moreover, although significant differences in fasting insulin could be observed, the result for each subject was reversed between initial visit and 1-year follow-up (13.50 ± 26.58 versus 12.57 ± 27.04, *p* = 0.010 and 14.43 ± 19.01 versus 17.10 ± 60.81, *p* = 0.002). The Bonnet index, the log-transform of HOMA-IR, was markedly elevated in the progression group compared with non-progression group (1.79 ± 0.65 versus 0.41 ± 0.56, *p* < 0.001 and 0.95 ± 0.84 versus 0.67 ± 0.95, *p* < 0.001). The lipid panel between two groups was similar while both the urine mAlb/Cr at index visit (12.12 ± 49.70 versus 3.65 ± 18.74, *p* = 0.011) and GFR (73.52 ± 25.48 versus 71.55 ± 23.33, *p* = 0.049) at the follow-up visit were significantly higher in the progression group. Table 3 shows index clinical presentations and angiography characteristics. No significant difference could be observed between the two groups.

**Table 2:** Comparison of laboratory results between the progression group and the non-progression group at first /follow-up visits

**Variable** **Initial/ follow-up visit**	**Progression Group (n = 198)**	**Non-progression Group (n = 168)**	*** p *value**
Fasting glucose (mmol/L)	6.15 ± 1.81	5.49 ± 1.25	<0.001
	7.84 ± 1.80	5.30 ± 1.22	0.005
OGTT (mmol/L)	10.09 ± 4.17	8.65 ± 3.41	<0.001
	9.68 ± 3.79	8.47 ± 3.38	0.002
Fasting insulin (mU/L)	13.50 ± 26.58	12.57 ± 27.04	0.010
	14.43 ± 19.01	17.10 ± 60.81	0.002
Postprandial insulin (mU/L)	78.31 ± 117.74	62.43 ± 65.86	0.388
	80.27 ± 71.31	88.35 ± 107.35	0.868
Bennett index	1.79 ± 0.65	0.41 ± 0.56	<0.001
	0.95 ± 0.84	0.67 ± 0.95	<0.001
HbA1c (%)	6.73 ± 1.40	6.24 ± 0.98	0.001
	6.79 ± 1.53	6.07 ± 0.78	<0.001
hsCRP (mg/L)	9.28 ± 13.56	13.34 ± 22.51	0.600
	6.55 ± 12.01	2.68 ± 4.14	0.048
BUN (mmol/L)	5.44 ± 1.59	5.45 ± 1.50	0.941
	6.06 ± 1.90	5.70 ± 1.48	0.623
Creatinine (umol/L)	84.47 ± 18.34	89.90 ± 36.51	0.671
	93.11 ± 23.46	87.28 ± 18.87	0.585
Urine acid (umol/L)	339.83 ± 76.41	339.30 ± 85.77	0.878
	358.38 ± 81.25	362.80 ± 79.00	0.966
Urine Ma/Cr	12.12 ± 49.70	3.65 ± 18.74	0.011
	16.00 ± 69.94	2.09 ± 3.14	0.366
GFR (ml/min)	80.16 ± 25.46	76.62 ± 28.98	0.345
	73.52 ± 25.48	71.55 ± 23.33	0.049
Serum triglyceride (mmol/L)	2.13 ± 1.72	1.86 ± 2.01	0.072
	1.76 ± 1.34	1.65 ± 1.50	0.207
Serum cholesterol (mmol/L)	4.58 ± 1.22	4.18 ± 1.09	0.103
	2.08 ± 0.76	2.05 ± 0.78	0.412
HDL (mmol/L)	1.05 ± 0.23	1.05 ± 0.26	0.706
	1.06 ± 0.26	1.14 ± 0.26	0.246
LDL (mmol/L)	2.81 ± 0.96	2.45 ± 0.84	0.118
	2.08 ± 0.76	2.05 ± 0.78	0.412
Lpa (mmol/L)	0.21 ± 0.21	0.20 ± 0.18	0.941
	0.21 ± 0.17	0.30 ± 0.49	0.317
ApoA (mmol/L)	1.13 ± 0.22	1.12 ± 0.20	0.764
	1.11 ± 0.22	1.12 ± 0.29	0.738
ApoB (mmol/L)	0.93 ± 0.28	0.87 ± 0.26	0.342
	0.80 ± 0.25	0.77 ± 0.23	0.225
Ejection Fraction	62.50 ± 9.06	64.01 ± 6.86	0.331
	62.62 ± 8.38	62.62 ± 8.20	0.153

**Table 3:** Index angiographic findings in the progression and non-progression groups

**Variables**	**Progression Group** **(N = 198)**	**Non-progression Group (N = 168)**	*** p *value**
Baseline Clinical presentation			
n (%)			
STEMI	49 (24.7)	44 (26.2)	0.752
NSTEMI	10 (5.1)	9 (5.4)	0.895
SAP	29 (14.6)	33 (19.6)	0.204
UAP	86 (43.4)	70 (41.7)	0.733
ECG abnormalities	24 (12.1)	12 (7.1)	0.111
Number of vessel affected n			0.332
(%)			
Without lesions	2 (1.0)	4 (2.4)	
Single vessel	50 (25.3)	56 (33.3)	
Double vessel	69 (34.8)	55 (32.7)	
Three vessel	74 (37.4)	51 (30.4)	
Multiple vessel	3 (1.5)	2 (1.2)	
Characteristics of lesion n			
(%)			
Diffuse lesion	51 (25.8)	53 (31.5)	0.221
Calcification	14 (7.1)	21 (12.5)	0.078
Bifurcation	13 (6.6)	16 (9.5)	0.296
Opening lesion	14 (7.1)	16 (9.5)	0.394
Occlusive lesion	37 (18.7)	33 (19.6)	0.817

We also obtained the changes in lab results by calculating the difference value between index and follow-up visits. Table 4 depicts the changes in lab results and their relationship with progression of atherosclerosis. During the course of the study, the glucose panel was elevated in the progression group compared with the non-progression group, including OGTT, which was significantly higher in progression group.

**Table 4:** Comparison of changes in lab results between the progression and non-progression groups

**Difference value of Variables**	**Progression group** **(n = 198)**	**Non-progression group** **(n = 168)**	*** p *value**
Glucose	0.21 ± 2.20	−0.19 ± 1.27	0.136
OGTT	−0.42 ± 3.92	−0.67 ± 2.87	0.025
Insulin	6.00 ± 23.46	2.42 ± 11.90	0.347
Insulin2h	20.75 ± 58.80	14.65 ± 49.81	0.200
SBP	−6.80 ± 21.76	2.68 ± 19.54	0.327
DBP	−5.65 ± 11.95	−1.58 ± 13.80	0.637
PP	−1.15 ± 18.76	4.26 ± 15.99	0.925
MAP	−6.03 ± 13.22	−0.16 ± 14.05	0.517
HbA1c	0.17 ± 1.28	0.02 ± 0.97	0.339
hsCRP	−4.61 ± 18.68	−11.19 ± 21.90	0.659
BUN	0.22 ± 1.39	0.48 ± 1.85	0.525
Creatinine	3.33 ± 16.63	4.28 ± 10.42	0.948
UA	20.25 ± 49.91	41.06 ± 70.41	0.311
GFR	−3.17 ± 12.38	−5.90 ± 14.05	0.520
Urine Malb/Cr	0.89 ± 66.12	0.20 ± 1.83	0.491
TG	−0.29 ± 0.91	−0.36 ± 1.58	0.279
TC	−0.90 ± 0.91	−0.57 ± 1.09	0.285
LDL	0.16 ± 0.43	0.16 ± 0.30	0.321
HDL	0.02 ± 0.22	0.06 ± 0.21	0.124
Lpa	−0.01 ± 0.09	0.14 ± 0.59	0.114
ApoA	0.02 ± 0.23	0.11 ± 0.25	0.919
ApoB	−0.09 ± 0.26	−0.08 ± 0.23	0.609
LVEF	−1.05 ± 5.65	−0.43 ± 5.83	0.561

We calculate the changes in laboratory results by deducting the index value from the follow-up value.

### Follow-up lab results, angiogram and Gensini score between the Higher Insulin Resistant group and Lower Insulin Resistant group

**Figure 2:**
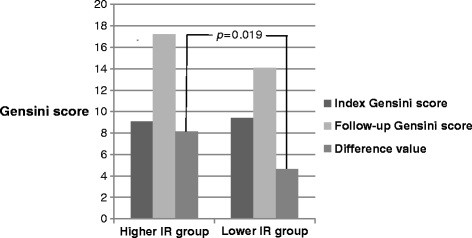
**Comparison of Gensini scores between the higher IR group and lower IR group at the initial/follow-up visits.*p*<0.05 was considered statistically significant.** Different colors represent different variables as listed on the right. Both index and follow-up Gensini score remained similar between the higher and lower IR groups (9.09 ± 14.33 versus 9.44 ± 12.88, *p* = 0.813 and 17.21 ± 18.46 versus 14.09 ± 14.18, *p* =0.358) while the difference value was significantly elevated in the higher than the lower IR group.

**Figure 3:**
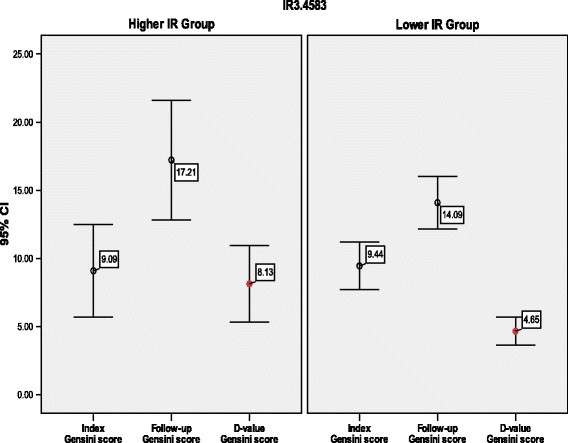
**Error bars demonstrating differences in index and follow-up Gensini score between the Higher and Lower IR group.** Each error bar represents a variable as listed on the X axis. The Y axis demonstrates the 95% confidence interval of each different Gensini score, including its mean value and standard deviation. The red circle indicates that difference value of Gensini score was statistically significant (8.13 ± 11.83 versus 4.65 ± 7.58, *p* = 0.019).

To investigate the IR further, we divided all participants into the higher IR group (71) and the lower IR group (213) based on the HOMA-IR cutoff value, which was 3.458 in our study. Table 5 presents the lab results from follow-up visits between two groups. Subjects in the higher IR group had markedly elevated laboratory values compared to the lower IR group, including BMI (27.69 ± 4.17 versus 24.13 ± 2.65, *p* < 0.001), prevalence of hypertension (84.5% versus 70.0%, *p* = 0.013), prevalence of diabetes (62.0% versus 33.8%, *p* < 0.001), fasting glucose (39.36 ± 9.93 versus 8.73 ± 3.43, *p* = 0.005), OGTT (10.51 ± 3.98 versus 8.73 ± 3.43, *p* = 0.002), fasting insulin (39.36 ± 96.93 versus 10.36 ± 9.56, *p* = 0.002), postprandial insulin (135.52 ± 148.57 versus 68.41 ± 60.23, *p* < 0.001), bonnet index (0.95 ± 0.84 versus 0.67 ± 0.95, *p* < 0.001) and HbA1c (6.79 ± 1.53 versus 6.07 ± 0.78, *p* < 0.001 ). In addition, laboratory results such as GFR (79.46 ± 28.19 versus 70.38 ± 21.72, *p* = 0.016), serum triglyceride (2.09 ± 1.75 versus 1.52 ± 1.01, *p* = 0.002) and hsCRP (8.54 ± 13.78 versus 3.87 ± 7.45, *p* = 0.005) were significantly elevated in the higher IR group compared to the lower IR group. Characteristics of follow-up angiograms demonstrated that the higher IR group had a markedly increased rate of plaque progression (65.7% versus 47.6, *p* = 0.009), new lesion in different vessels (47.1% versus 27.4%, *p* = 0.002) and exacerbation of original lesion (22.9% versus 9.0%, *p* = 0.002) than in the lower IR group (Table 6). Moreover, while the initial and follow-up Gensini scores were similar between these two groups (9.09 ± 14.33 versus 9.44 ± 12.88, *p* = 0.813 and 17.21 ± 18.46 versus 14.09 ± 14.18, *p* =0.358), the difference value during the follow-up is markedly elevated in the higher IR group than the lower IR group (8.13 ± 11.83 versus 4.65 ± 7.58, *p* = 0.019) (Figures 2 and 3).

**Table 5:** Comparison of lab results between the higher IR and lower IR groups (only variables considered statistically significant were listed)

**Variables** **At the follow-up visit**	**Higher IR group** **(n = 71)**	**Lower IR group (n = 213)**	*** p *value**
BMI	27.69 ± 4.17	24.13 ± 2.65	<0.001
Prevalence of hypertension	60 (84.5%)	148 (70.0%)	0.013
Prevalence of diabetes	44 (62.0%)	72 (33.8%)	<0.001
Fasting glucose	7.84 ± 1.80	5.30 ± 1.22	0.005
OGTT	10.51 ± 3.98	8.73 ± 3.43	0.002
Fasting insulin	39.36 ± 96.93	10.36 ± 9.56	0.002
Postrandial insulin	135.52 ± 148.57	68.41 ± 60.23	<0.001
Bonnet index	0.95 ± 0.84	0.67 ± 0.95	<0.001
HbA1c	6.79 ± 1.53	6.07 ± 0.78	<0.001
GFR	79.46 ± 28.19	70.38 ± 21.72	0.016
Triglycerides	2.09 ± 1.75	1.52 ± 1.01	0.002
hsCRP	8.54 ± 13.78	3.87 ± 7.45	0.005

We divided the subjects into higher IR group and lower IR group based on the cutoff value, defined as the highest quartile of HOMA-IR from 366 participants and equaled to 3.458 in our study.

**Table 6:** Comparison of the Gensini score and the pattern of follow-up angiograms between the higher IR and lower IR groups

	**Higher IR group** **(n = 71)**	**Lower IR group (n = 213)**	*** p *value**
Initial gensini score	9.09 ± 14.33	9.44 ± 12.88	0.813
Follow-up gensini score	17.21 ± 18.46	14.09 ± 14.18	0.358
Difference value during	8.13 ± 11.83	4.65 ± 7.58	0.019
1-year peroid			
Follow-up angiogram (%)			
Progression of plaques	46(65.7)	101(47.6)	0.009
Revascularization	28 (40.6)	77(36.7)	0.561
New lesion in different	33 (47.1)	58 (27.4)	0.002
vessel			
Exacerbation of original	16 (22.9)	19 (9.0)	0.002
lesion			
In-stent restenosis	13(18.3)	31(14.6)	0.449

### Multivariable regression analysis of related risk factors towards atherosclerotic progression

We assessed the impact of conventional and novel risk factors on the coronary atherosclerosis progression by using a multivariate logistic regression analysis (Table 7). Risk factors including age, sex, BMI, prevalence of hypertension or diabetes, HOMA-IR > 3.458, HbA1c, hsCRP, LDL-C, urine MA/Cr and change in OGTT were entered in the model and the results revealed that both HOMA-IR > 3.458(OR = 4.969, *p* = 0.010) and HbA1c (OR = 1.721, *p* = 0.034) were independent predictors of progression of coronary lesions.

**Table 7:** Regression analysis of risk factors for plaque progression concerning all participants

**Variable**	**OR value**	**95%CI**	*** p *value**
HOMA-IRI > 3.458	4.969	0.630-6.475	0.011
HbA1c	1.721	0.256-4.515	0.034

Abbreviations: CI, confidence interval; HbA1c, glycosylated hemoglobin; HOMA-IRI, homeostasis model assessment insulin resistance index; hsCRP, high sensitive C - reactive protein; OR, odds ratio.

Binomial regression analysis was performed across the entire study group. Regression model included both conventional and novel risk factors for coronary plaque progression such as age, sex, body mass index, hypertension, diabetes mellitus, follow-up urine microalbumin/creatinine, follow-up hsCRP, follow-up HbA1c, follow-up LDL. Change in OGTT was also included.

We then divided all subjects into the diabetic (n = 136) and non-diabetic groups (n = 230) to investigate the role of insulin resistance in the development of atherosclerotic plaques separately. The same binomial regression models were set up except the input of prevalence of diabetes mellitus (Table 8). Insulin resistance remained an independent predictor for progression of coronary lesions in both groups according to the result. In addition, because HOMA-IR could be modeled as a linear continuous variable or a categorical variable divided by its cutoff value [12,13], we subsequently tested HOMA-IR from both perspectives. The result revealed that HOMA-IR was an independent predictor of atherosclerotic progression, which was consistent with our early findings.

**Table 8:** Logistic Regression of Risk Factors in both Diabetic Participants and non-Diabetic Participants

**Subgroups**	**Variable**	**OR value**	**95%CI**	*** p *value**
Non-DM	HOMA-IRI > 3.458	7.528	0.973-4.300	0.034
DM	HOMA-IRI > 3.458	7.590	0.987-4.220	0.040

Abbreviations: CI, confidence interval; DM, diabetes mellitus; HbA1c, glycosylated hemoglobin; HOMA-IRI, homeostasis model assessment insulin resistance index; hsCRP, high sensitive c-reactive protein; OR, odds ratio.

Binomial regression was performed separately in the non-DM (n = 230) and DM groups (n = 136). The model included both conventional and novel risk factors for coronary plaque progression such as age, sex, body mass index, hypertension, follow-up urine microalbumin/creatinine, follow-up hsCRP, follow-up HbA1c, follow-up LDL.

## Discussion

Our study explored the effect of insulin resistance on the progression of atherosclerotic plaques over a 1-year follow-up interval. The difference value of Gensini score between the higher IR group and the lower IR group was statistically significant and the binomial multivariate regression models contended that insulin resistance was an independent predictor of atherosclerotic progression in patients with coronary heart disease irrespective of the individual’s diabetes status. Insulin resistance generally occurs in cluster with other risk factors including hyperglycemia, dyslipidemia and inflammatory conditions. IR itself is a phenomenon that decreases the effects produced by the normal activity of insulin [13]. In addition, one of its key biochemical defects lies in the specific impairment of PI3K-dependent signaling pathways while other pathways including MAPK were intact [14], which results in the overproduction of ET-1 over eNOS, leading to endothelium dysfunction and enhanced levels of VCAM-1 and MCP-1, eventually contributing to the formation of atherosclerotic plaques [15]. Early studies have already supported the idea that IR is an important predictor of CHD [16]. Tetsuya conducted a prospective study and found that IR is associated with coronary lipid-rich plaques in patients with abnormal glucose regulation [17]. Reports have also indicated that IL-6, a potential cardiometabolic biomarker expressing in human atherosclerotic lesions, has a close relationship with HOMA-IR [18]. Uli C argues that low adiponectin levels are frequently associated with insulin resistance, may have a closer link with coronary plaque vulnerability, which plays an important role in the pathogenesis of ACS [19]. Having enrolled 543 patients with diabetes and CHD and using the intravascular ultrasound (IVUS) technique to assess the progression of coronary atherosclerosis directly, the recent PERISCOPE study concluded that pioglitazone, a drug targeting IR, could significantly slow the progression of atherosclerosis, which is also supportive of our study [20].

Having confirmed the predictive value of IR on plaques progression, our study also looks into the issue of in-stent restenosis, an important complication of coronary arterial intervention nowadays [21]. Having observed similar prevalence of in-stent restenosis between the higher and lower IR groups, we set up the binary logistic regression model to evaluate the risk factors for in-stent restenosis by inputting the same variables as above. The results showed that unlike prevalence of HTN (OR = 0.241, *p* = 0.019) and age (OR = 1.084, *p* = 0.008), insulin resistance had no statistically significant effect on the development of in-stent restenosis (OR = 2.064, *p* = 0.218, table not shown). It has been well studied that the formation of in-stent restenosis depends on the proliferation of in-stent neointimal cells because of complex inflammatory responses in the short time interval, which is quite different from the process of plaque formation [21,22]. This may serve to explain the results of our study. However, conflicting evidence does exist. A study in 2005 by Kazuaki stated that IR predicted in-stent restenosis rather than de-novo stenosis, which was mediated by atherosclerosis plaque progression [7]. Following the difference can be utilized to explain the differences in results between their study and ours according to the following facts: 1. the aim of the study by Kazuaki was to evaluate the IR’s effect on restenosis after stenting, which resulted in the 110 recruited individuals were not just confined to subjects with CHD. Instead, the sample included patients who had extensive coronary artery dissection after PTCA, complete vessel closure, residual stenosis of 25% or more of the vessel diameter. 2. The follow-up interval in Kazuaki’s study, which played an important role in the development of in-stent restenosis was 4-months. In our case, the interval was 1 year. 3. Instead of setting up a cutting value for HOMA-IR to determine the state of insulin resistance as we did, they set the HOMA-IR as a continuous variable. 4. The variables they used in the multivariable logistic regression model only included HbA1C, HOMA-IR and LDL-C, which in our case are risk factors such as age, sex, BMI, prevalence of hypertension and diabetes, HOMA-IR, HbA1c, hsCRP, LDL-C and urine MA/Cr. Based on these differences in the fundamental aspects of the studies, it seems inappropriate to compare their results with ours.

Our study also revealed that HbA1c also stands for an independent predictor of coronary plaque progression in unselected individuals. As a reliable marker of recent 2–3 months serum glucose level, HbA1c has already been used for diagnosing diabetes mellitus [23,24]. In a recent meta-analysis covering 20 studies involving 13, 224 individuals suggested that HbA1c level was an independent risk factor for mortality in CHD patients without diabetes [25]. Hiroyasu also stated that HbA1c was an independent predictor of major adverse cardiac events after the implantation of DES [26]. Several other studies also support the role of HbA1c in future CVD event and mortalities [27-29].

### Study limitations

First, the number of subjects recruited in the study was limited and all individuals were enrolled from a single center at the Shanghai Ruijin hospital. In addition, the majority of patients originated from Shanghai, Jiangsu province and Zhejiang province, all confined to the southeast region of the Yangzi River in Mainland China. Moreover, due to the limited number of individuals in subgroups such as the IGT group (n = 57) and the IFG group (n = 39), our study did not perform the subgroup analysis and future studies may provide different results, as the pathogenesis of IGT is different from that of IFG concerning insulin resistance [30]. Finally, discordant views did exist in assessing insulin resistance by using the HOMA-IR method, including the lack of consensus on setting up a unified cutoff value for HOMA-IR to determine the state of IR [31]. We chose to set the upper quartile of HOMA-IR in the background population as its cutoff value [8,10], which equaled 3.458 in our study. However, we also modeled HOMA-IR as a linear continuous variable and tested it from both perspectives in our regression model, and the results conformed to each other.

## Conclusions

Insulin resistance is an independent predictor for atherosclerosis plaque progression in patients with coronary heart disease in both the diabetic and non-diabetic population.

## Abbreviations

ACS, Acute coronary syndrome; AMI, Acute myocardial infarction; BMI, Body mass index; CABG, Coronary artery bypass graft; CI, Confidence interval; DM, Diabetic mellitus; eNOS, Enzyme nitric oxide synthase; ET-1, Endothelin-1; FIN, Fasting insulin; FPG, Fasting plasma glucose; GFR, Glomerular Filtration Rate; HbA1c, Glycosylated hemoglobin; HDL, High-density lipoprotein; HR, Hazard ratio; HIS, Hyperinsulinemia; HOMA-IRI, Homeostasis model assessment insulin resistance index; hsCRP, High sensitive c-reactive protein; ICAM-1, Intercellular adhesion molecule-1; IFG, Impaired fasting glucose; IGT, Impaired glucose tolerance; IL-6, Interleukin-6; IR, Insulin resistance; LDL, Low-density lipoprotein; LDL-C, Low-density lipoprotein-cholesterol; Lpa, Lipoprotein (a); Lp-PLA2, Lipoprotein-associated phospholipase A2; LVEF, Left ventricular ejection fraction; MCP-1, Monocyte chemotactic protein 1; NCD, Non-communicable Disease; NGR, Normal glucose regulation; NSTEMI, Non ST-segment elevation myocardial infarction; NO, Nitric oxide; OGTT, Oral glucose tolerance test; PAI-1, Plasminogen activator inhibitor-1; PCI, Percutaneous coronary intervention; PI3K, Phosphatidylinositol 3-kinase; PTCA, Percutaneous transluminal coronary angioplasty; SD, Standard deviation; STEMI, ST-segment elevation myocardial infarction; TC, Total cholesterol; TG, Triglyceride; TIMI, Thrombolysis in myocardial infarction; TNF-α, Tumor Necrotizing Factor-α; UAP, Unstable angina pectoris; VCAM-1, Vascular cell adhesion molecule; VSMC, Vascular smooth muscle cells.

## Competing interests

The authors of the present study declare that they have no competing interests.

## Authors’ contribution

Xuanqi An, the first author, has completed the entire paper based on data collected from Jan 2007 to Dec 2008 and from Jan 2010 to December 2011. Dong Yu collected the data during the year 2009. Ruiyan Zhang, our correspondence author and the director of the catheter lab at the Shanghai Ruijin hospital, has contributed enormously to devising the entire study plan and supervising the entire process. Jingzhou Zhu helped greatly with editing the presented manuscript. Other authors contributed equally. All authors read and approved the final manuscript.
